# Expression of *ERG11*, *ERG3*, *MDR1* and *CDR1* genes in *Candida tropicalis*

**DOI:** 10.7705/biomedica.6852

**Published:** 2023-08-31

**Authors:** Ana Elisa Rojas, Leidy Yurany Cárdenas, María Camila García, Jorge Enrique Pérez

**Affiliations:** 1 Grupo de Investigación en Enfermedades Infecciosas - GINEI, Universidad Católica de Manizales, Manizales, Colombia. Universidad Católica de Manizales Universidad Católica de Manizales Manizales Colombia; 2 Grupo de Investigación en Enfermería - GRIEN, Universidad Católica de Manizales y Universidad de Caldas, Manizales, Colombia. Universidad Católica de Manizales Universidad Católica de Manizales Universidad de Caldas Manizales Colombia; 3 Grupo de Investigación BIOSALUD, Universidad de Caldas, Manizales, Colombia. Universidad de Caldas Universidad de Caldas Manizales Colombia

**Keywords:** Candida tropicalis, drug resistance, fungal, fluconazole, Candida tropicalis, farmacorresistencia fúngica, fluconazol

## Abstract

**Introduction.:**

Drug resistance to azoles is a growing problem in the *Candida* genus.

**Objective.:**

To analyze molecularly the genes responsible for fluconazole resistance in *Candida tropicalis* strains.

**Materials and methods.:**

Nineteen strains, with and without exposure to fluconazole, were selected for this study. The expression of *MDR1*, *CDR1*, *ERG11*, and *ERG3* genes was analyzed in sensitive, dose-dependent sensitive, and resistant strains exposed to different concentrations of the antifungal drug.

**Results.:**

*MDR1*, *ERG11* and *ERG3* genes were significantly overexpressed in the different sensitivity groups. *CDR1* gene expression was not statistically significant among the studied groups. Seven of the eight fluconazole-resistant strains showed overexpression of one or more of the analyzed genes. In some dose-dependent sensitive strains, we found overexpression of *CDR1*, *ERG11*, and *ERG3*.

**Conclusion.:**

The frequency of overexpression of *ERG11* and *ERG3* genes indicates that they are related to resistance. However, the finding of dose-dependent resistant/sensitive strains without overexpression of these genes suggests that they are not exclusive to this phenomenon. More basic research is needed to study other potentially involved genes in the resistance mechanism to fluconazole.

## Resumen

**Introducción.:**

La farmacorresistencia a los azoles es un problema creciente en el género *Candida*.

**Objetivo.:**

Analizar molecularmente los genes responsables de la resistencia a fluconazol en cepas de *Candida tropicalis*.

**Materiales y métodos.:**

Para este estudio, se seleccionaron 19 cepas, con exposición a fluconazol y sin ella. Se analizó la expresión de los genes *MDR1*, *CDR1*, *ERG11* y *ERG3* en cepas sensibles, sensibles dependiente de la dosis, y resistentes, previamente expuestas a diferentes concentraciones del fármaco antifúngico.

**Resultados.:**

Se encontró que los genes *MDR1*, *ERG11* y *ERG3* estaban significativamente sobreexpresados en los diferentes grupos de sensibilidad. La expresión del gen *CDR1* no fue estadísticamente significativa entre los grupos estudiados. Siete de las ocho cepas resistentes a fluconazol mostraron sobreexpresión de uno o más de los genes analizados. En algunas cepas sensibles dependientes de la dosis, se encontró sobreexpresión de *CDR1*, *ERG11* y *ERG3*.

**Conclusión.:**

La sobreexpresión de los genes *ERG11* y *ERG3* indica que están relacionados con la resistencia de las cepas de *Candida*. Sin embargo, el hallazgo de cepas resistentes o sensibles según la dosis, sin sobreexpresión de estos genes, sugiere que pueden existir otros genes involucrados en este fenómeno. Se necesitan más investigaciones básicas que contribuyan al estudio de otros genes potencialmente involucrados en el mecanismo de resistencia al fluconazol.

Different *Candida* species are associated with infections involving mucous membranes, spreading to the bloodstream and even reaching deep tissues ^1^. In particular, *Candida tropicalis* has been related to infections in patients with neutropenia or those in intensive care units ^2^. It is recognized as the etiologic agent of acute cutaneous candidiasis in neutropenic patients, and some studies have reported that deep infections caused by this species are usually more lethal than those caused by other non*albicans* species ^3^.

Currently, there are some antifungal treatment options for *Candida* spp. infections. Azoles, polyenes, and echinocandins are among the most widely used pharmacological groups ^1^^,^^4^. The azoles, especially fluconazole, are extensively applied in prophylaxis and direct intervention in patients with *Candida* spp. infections. In fact, some reports relate the frequent use of this pharmacological group with the acquired resistance ^5^.

The azoles are broad-spectrum fungistatic compounds whose primary mechanism of action, after entering the yeast, is its interaction with the enzyme lanosterol 14-alpha-demethylase. This interaction decreases the enzyme affinity for the endogenous substrate, lanosterol. As a result, there is a blockage of the signaling pathway involved in ergosterol synthesis, which is the main lipid component of the *Candida*’s membrane ^6^^,^^7^.

A worldwide problem has emerged around *Candida* spp. infections and their pharmacological intervention: antifungal resistance. The definition of resistance is the nonsusceptibility of the fungus to the drug or pharmacological group of intervention. It can be of two types: primary (intrinsic) or secondary (acquired) ^5^. This resistance undermines the efficacy of the pharmacological intervention and is associated with therapeutic failure. Thus, the use of some drugs, such as fluconazole, has been controversial, not only because of reported resistance but also because it contributes to generate. Greater resistance, complications and waste of healthcare resources ^8^.

It has been reported that *C. tropicalis* shows significant azole resistance, with high resistance to fluconazole compared to *C. albicans* and *C. parapsilosis*^9^. According to reported data by the SENTRY antifungal surveillance program, worldwide resistance of *C. tropicalis* to fluconazole ranges from 2.5% to 4.9%, with rates above 9% in the Asia-Pacific region ^10^. The main factors involved in resistance development are decreased concentrations of the drug inside the yeast and reduced affinity with lanosterol 14-alpha-demethylase ^11^. It is common to find that these resistance mechanisms are associated with alterations in gene expression or mutations and deletions in particular genes. For example, *CDR1* and *MDR1* encoding proteins of the ABC family (Adenosine Triphosphate Binding Cassette) and the Major Facilitator Superfamily (MFS) superfamily, involved in the transport and exit of fluconazole through the yeast plasma membrane, have been widely reported to be overexpressed ^12^. Overexpression or mutations of the *ERG11* gene coding for the enzyme lanosterol 14-alpha-demethylase have also been identified in resistant strains, leading to a lower affinity between fluconazole and its pharmacological target ^13^^,^^14^. Another molecular mechanism associated with fluconazole resistance expressed by *C. tropicalis* involves mutations in the *ERG3* gene, which encodes the enzyme sterol 5,6demethylase, responsible for converting non-toxic intermediate sterols - derived from the pharmacological action of fluconazole - into toxic sterols ^13^. These mutations cause a reduction of the aforementioned toxic intermediates in the yeast cell membrane, with the consequent inability to cause fungal death ^8^. The main mechanism described in the literature, justifying the resistance of *C. tropicalis* to azoles, corresponds to drug concentrations decrease inside the fungus by overexpression of efflux or extrusion pumps. However, other related mechanisms can configure resistance in isolation or simultaneously occur, such as mutations in the *ERG11* and *ERG3* genes ^11^.

In a previous study, conducted in an intensive care unit of a hospital in the city of Manizales, the frequency of *Candida* spp. colonization was detected in admitted patients. The authors found a high frequency of colonization with different *Candida* species, being *C. tropicalis* the third in frequency after *C. albicans* and *C. glabrata* (unpublished data) ^15^. Given the high frequency of colonization by *C. tropicalis*, the aim of this study was to analyze the relative expression of *MDR1*, *CDR1*, *ERG11*, and *ERG3* genes in strains exposed/ not-exposed to fluconazole according to their antifungal sensitivity profile.

## Materials and methods

### Sample selection and group analysis

In the performed gene expression experiments, we followed the guidelines proposed in the Minimum Information for Publication of Quantitative RealTime PCR Experiments (MIQE) ^16^. Nineteen strains of *C. tropicalis* from a previous investigation entitled “Study of the colonization of *Candida* species in older adults at intensive care admission” were selected. The strains were plated on potato dextrose agar (PDA, Scharlau Microbiology), and their identification was performed on the Vitek 2 compact device (Biomerieux). Each strain was tested for antifungal susceptibility using the protocols proposed by *The Clinical and Laboratory Standards Institute* (CLSI), version M27-A3 ^17^. According to the obtained results, the strains were classified into three susceptibility groups: sensitive with a minimum inhibitory concentration ≤ 2μg/ml, dose-dependent sensitive with a minimum inhibitory concentration of 4 μg/ml, and resistant with minimum inhibitory concentration ≥ 8 μg/m.

### Macrodilution assay with and without fluconazole

Each strain was subjected to a macrodilution assay according to the protocol proposed by CLSI (M27-A3), and liquid Sabouraud was used as a culture medium. All tubes were incubated at 35 °C until reaching the yeast logarithmic growth phase. At the time of the minimum inhibitory concentration assessment, the size of the cell sediment of each fluconazole concentration was compared with the positive control. Cells were collected from the tube when growth was higher than 50%. The cell concentration was adjusted to a value between 2-3 x 10^8^ blastoconidia per ml for all the samples and the control. The tubes were centrifuged, and the yeast sediment was stored in Eppendorf tubes, free of DNAse and RNAse, containing RNA later (RNAaseZap solution, Ambion RNA technology), and frozen at -80 °C until the moment of RNA extraction.

### RNA extraction

To obtain RNA, we followed the protocol proposed in the RiboPure-Yeast Kit (Ambion RNA technology) according to the manufacturer’s recommendations.

### RNA extraction quality criteria

The RNA samples were subjected to 1% agarose gel electrophoresis to observe the integrity of the genetic material. The RNA was considered not degraded when two bands appeared on the gel. RNA purity was measured using the UVIS Drop UVS99™ spectrophotometer (Avans Biotechnology) with absorbance readings at 230, 260, and 280 nm. Those RNAs with a 260:280 and 260:230 ratio higher than or equal to 2, were of high purity and were used in RT-qPCR assays.

To evaluate the presence of DNA after treatment with DNase I, we performed PCR amplification of one RNA sample, without reverse transcription, using an universal primer for *C. tropicalis*. The DNA- contaminated strains were subjected to a new DNase I treatment.

### Protocol standardization

The *C. tropicalis* strain from the American Type Culture Collection, ATCC750 was used as a sensitive strain reference, and a strain from the fluconazole-resistant group was randomly selected as resistance reference. All procedures were performed according to previously established guidelines to ensure adequate purity of the reactions and to avoid contamination with RNA from other microorganisms.

### Reverse transcription protocol

The reverse transcription process was performed in the StepOnePlus^TM^ Real-Time PCR System thermocycler (Applied Biosystems), with the following protocol: Incubation at 25 °C for 10 minutes, then at 42 °C for 15 minutes, and finally, the enzyme was inactivated at 85 °C for 5 minutes. The obtained RNA was converted to cDNA using the SensiFast^TM^ cDNA kit (Bioline). The manufacturer’s instructions were carefully followed, with no modifications.

### Selection and optimization of primers for qPCR

The primer sequences for *MDR1* and *CDR1* amplification were proposed by Jiang *et al*. ^18^, and the primer sequences for *ACT1*, *ERG11*, and *ERG3* amplification were designed in the laboratory (table 1). The algorithm used to generate the primers was the OligoPerfect Primer Design^TM^ (Thermo Fisher), and the reference sequence corresponded to *C. tropicalis* MYA3404 (GenBank accession number XM_002550136 - National Center for Biotechnology of information - NCBI). The ideal alignment temperatures were selected by melting curve analysis, ensuring the specificity of the evaluated primers.

**Table 1:** First sequence

Gene		Primer sequence (5'-3')	Size (bp)	Melting temperature (Tm) (°C)
*ACT1*	Forward	GTGTTACCCACGTTGTCCCA	139	81,42
	Reverse	GCGGTGGTGGAGAAAGTGTA		
*MDR1*	Forward	TAAAGCAGGCTGGAGATGGA	444	78,11
	Reverse	ACAACCTCCAACTATAGCTA		
*CDR1*	Forward	TGAAGCCAGACCCGTAGTTG	379	78,22
	Reverse	CCACTTTGCCCATCCTAACA		
*ERG11*	Forward	CCATGGTTTGGTCTGCTGC	140	75,86
	Reverse	TCGTGACCTTTTGGACCCA		
*ERG3*	Forward	TGGAAATCGGTTTGGCAACT	149	76,26
	Reverse	AGGAAATTGCCATAAAAGTGCCT		

### qPCR experiment

Each qPCR assay with the samples exposed or non-exposed to fluconazole was performed in triplicate (a replicate technique to monitor the precision of real-time PCR amplification) on the StepOnePlus^TM^ RealTime PCR machine using the PowerUp SYBR Green Master Mix kit (Applied Biosystems). *ACT1* was used as a reference gene to evaluate the transcriptional relative expression of the *MDR1*, *CDR1*, *ERG11*, and *ERG3* genes. The specificity of the products of q-PCR reactions was evaluated through dissociation curves analysis (melting curve).

Each q-PCR quantification reaction contained 5 μΙ of 2X PowerUp SYBR Green Master Mix, 0.8 μl of each primer at a final concentration of 0,8 μΜ, 1 μl of cDNA (cDNA adjusted concentration to 1 ng/μl), the volume was made up to 10 μl with RNAse/DNAse-free water. The program consisted of an initial denaturation in two steps: 50 °C for two minutes and 95 °C for 10 minutes, followed by 40 cycles of denaturation at 95 °C for 15 seconds and an extension at 60 °C for 1 minute. To establish the cDNA dissociation temperature and identify non-specific amplification reactions, the following parameters were used: 95 °C for 15 seconds, 60 °C for 1 minute, and 95 °C for 15 seconds.

### Relative expression assessment of MDR1, CDR1, ERG11 and ERG3

The relative expression was calculated using the difference between the amplification cycle (Ct) of the resistance genes (*MDR1*, *CDR1*, *ERG11*, *ERG3*) and the reference endogenous control gene (*ACT1*), per sample. *C. tropicalis* ATCC-750 was used as the reference strain. Analysis of the results was performed in StepOne Plus^TM^ Real-Time PCR software, applying double-delta Ct equation (2-ΔΔCT) (Applied Biosystems). This same software allowed efficiency calculation of each evaluated reaction.

### Statistical analysis

Data normality Kolmogorov-Smirnov test was performed, and according to the result, we used t Student for parametric data and Wilcoxon test for non-parametric data. These statistical analyses were applied to establish gene expression significance when comparing the samples in two conditions: Exposed to fluconazole (test group) and notexposed to fluconazole (control group). A value of p < 0.05 was considered statistically significant.

## Results

The relative expression of the studied genes of the 19 strains exposed and not-exposed to the antifungal indicated that *MDR1*, *ERG11*, and ERG3 expression in the fluconazole-exposed strains was higher compared to the unexposed strains expression. However, the greatest increase in the relative expression of the exposed *versus* unexposed strains corresponded to *ERG11* (fold change of 2.48, p = 0.009), followed by *MDR1* (fold change of 1.69, p = 0.099), and finally *ERG3* (fold change of 1.35, p = 0.0045). On the contrary, the *CDR1* gene in fluconazole-exposed strains showed a lower relative expression than those not-exposed (fold change of -0.22, p = 0.035).

Of the 19 strains tested, 8 were resistant (samples: 68-1, 100-1, 132-2, 48-2, 23-1, 95-1, 176-1, 103-1), 6 were dose-dependent sensitive (samples: 84-1, 46-1, 109-1, 68-2, 2-2B, 159-1) and 5 were sensitive (samples: 105-1, 108-1, 156-1, 135-1, 85-1). Here, we described the findings obtained when comparing the relative expressions of the ATCC reference strain, exposed and not-exposed to fluconazole, with different strains according to their fluconazole sensitivity profile.

**Figure 1:**
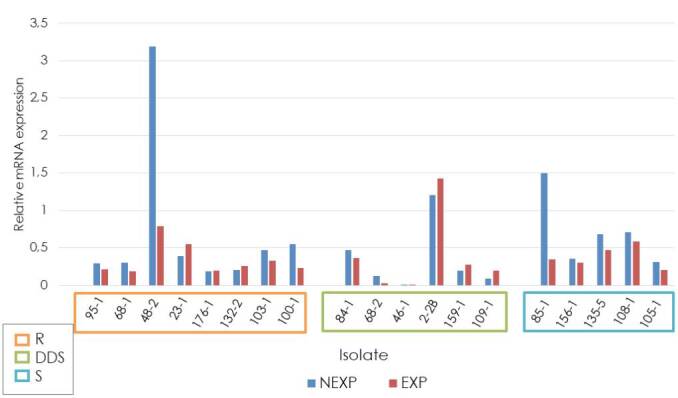
Relative expression of the CDR1 gene in *Candida tropicalis*

When analyzing the expression of the *CDR1* gene, we found that ATCC reference strain relative expression (fold change of 1) was exceeded by the resistant strain 48-2 (notexposed to fluconazole), by the dose-dependent sensitive strain 2-2B (exposed and not-exposed to fluconazole), and by the sensitive strain 85-1 (not-exposed to fluconazole). The average relative expression of the *CDR1* gene in the other analyzed strains did not exceed the relative expression of the ATCC reference strain. The relative expression was higher in strains exposed to fluconazole compared to non-exposed strains in the dose-dependent sensitive group. However, this difference was not statistically significant (p = 0.275). Concerning relative expression in the resistant and sensitive groups, we found lower expression in those exposed to fluconazole in both groups (figure 1).

R: Resistant; DDS: Dose-dependent sensitive; S: Sensitive; NEXP: Not-exposed to fluconazole; EXP: Exposed to fluconazole

**Figure 2:**
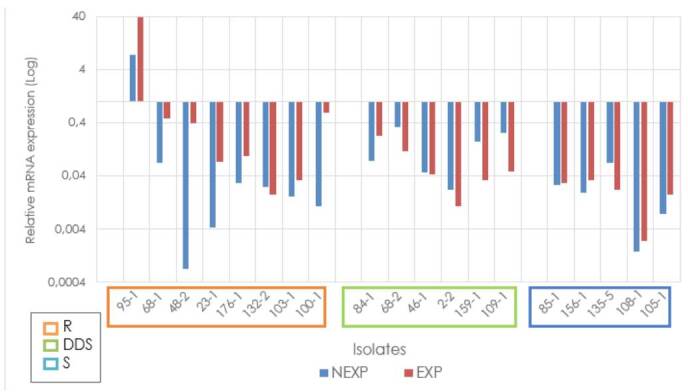
Relative expression of the *MDR1* gene in *Candida tropicalis*

In general, the average relative expression of the *MDR1* gene of the antifungalexposed and not-exposed strains did not exceed the relative expression of the ATCC reference strain. However, overexpression of this gene was observed in one of the strains fluconazole-exposed and resistant to it (95-1) with a fold change higher than 30. Additionally, it was found that strains exposed and resistant to fluconazole had a higher relative expression than those not-exposed (fold change of 4.08, p= 0.0085). Regarding the groups classified as sensitive and dose-dependent sensitive, we identified that strains exposed to fluconazole had a lower relative expression than nonexposed strains (figure 2).

R: Resistant; DDS: Dose-dependent sensitive; S: Sensitive; NEXP: Not-exposed to fluconazole; EXP: Exposed to fluconazole **Note:** Due to the extreme values of the relative expression of some isolates, it is graphed on a logarithmic scale to facilitate visualization.

**Figure 3:**
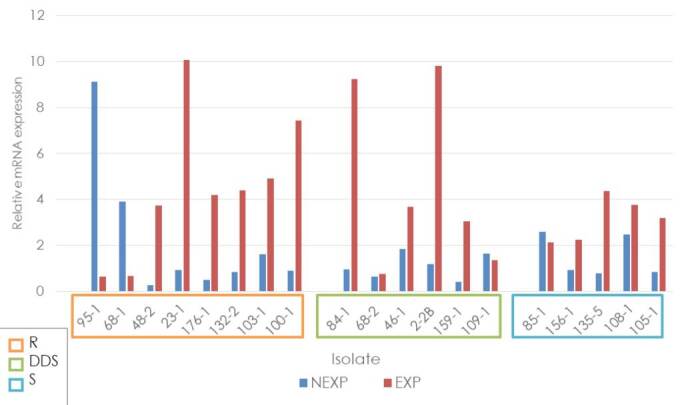
Relative expression of the *ERG11* gene in *Candida tropicalis*

The average relative expression of the *ERG11* gene, independent of the classification according to the fluconazole sensitivity profile and the condition of exposure or not to the antifungal, always exceeded the relative expression of the ATCC reference strain. Six of the eight samples exposed and resistant to fluconazole showed overexpression with a fold change higher than 3 (23-1, 482, 100-1, 103-1, 132-2, and 176-1). In contrast, in the dose-dependent sensitive group, four of the six samples showed overexpression higher than 2 (2-2, 46-1, 84-1, and 159-1), and in the sensitive group, four of the five samples showed values between 2 and 4 (105-1, 108-1, 135-5 and 156-1) (figure 3).

R: Resistant; DDS: Dose-dependent sensitive; S: Sensitive; NEXP: Not-exposed to fluconazole; EXP: Exposed to fluconazole

The highest average relative expression was observed in the exposed strains from the dose-dependent resistant and dose-dependent sensitive groups. There was a higher relative expression in fluconazole-exposed strains compared with those not-exposed in all groups classified according to the sensitivity profile (resistant group fold change = 2.24, p = 0.14; dosedependent sensitive group fold change = 3.53, p = 0.035; and sensitive group fold change 1.61, p = 0.036).

**Figure 4:**
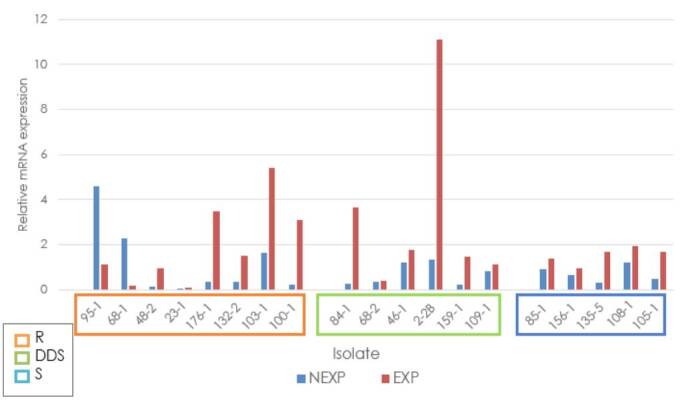
Relative expression of the *ERG3* gene in *Candida tropicalis*

Independent of the antifungal sensitivity profile classification, the average relative expression of the ERG3 gene in fluconazole-exposed strains always exceeded the relative expression of the ATCC reference strain. Among the exposed resistant strains, 103-1 showed overexpression with a fold change of 4, while strains 100-1, 132-2, and 176-1 showed a fold change between 1 and 4. In the dose-dependent sensitive strains, overexpression was identified in 2-2B with a fold change higher than 10, and in strains 461, 84-1, 109-1, and 159-1, fold changes were between 1 and 4. In the sensitive group, four samples had fold changes between 1 and 2 (85-1, 105-1, 108-1, and 135-5), and one was below 1 (156-1) (figure 4).

R: Resistant; DDS: Dose-dependent Sensitive; S: Sensitive; NEXP: Not-exposed to fluconazole; EXP: Exposed to fluconazole

There was a higher relative expression in fluconazole-exposed strains in all groups of analysis classified according to the sensitivity profile in comparison with the notexposed: resistant group fold change = 0.78, p = 0.20; dose-dependent sensitive group fold change = 2.55, p = 0.014; sensitive group fold change = 0.81, p = 0.0085).

From the group of strains classified as resistant, seven samples overexpressed at least one gene (48-1, 95-1, 23-1, 100-1, 103-1, 132-2, and 176-1), and two of these seven overexpressed two or more genes (100-1 and 103-1); in the dose-dependent sensitive strains, two samples overexpressed at least two genes (2-2 and 84-1). Strain 95-1 exposed to fluconazole showed a much higher expression of the *MDR1* gene but not of the other genes analyzed in this study.

## Discussion

Given the growing importance of the azole resistance phenomenon in *Candida* spp., multiple studies worldwide have focused their interest on basic research intending to broaden and deepen the theoretical reference, which allows taking elements of analysis with clinical projection. Research has been conducted on resistance, especially in *C. albicans*. In Colombia, this would be the first study on resistance mechanisms in the species *C. tropicalis*, the study of which has recently focused on resistance ^13^^,^^19^^-^^21^. In the region, researchers published a study about the identification and frequency of molecular mechanisms associated with fluconazole resistance expressed by *C. albicans* species ^22^. However, similar studies have not been reported for *C. tropicalis*, an etiologic agent frequently isolated from infections and, according to some studies, causative of higher mortality ^23^.

The results of this study indicate that fluconazole-exposed strains, independent of the sensitivity profile, had a higher expression of the *ERG11*, followed by *ERG3* and *MDR1*. These results could suggest that the drugstimuli exposure could lead to biological modifications in *C. tropicalis* strains, at the gene expression level, affecting enzymesencoding genes more than transporters-encoding genes potencially involved in drug extrusion; a similar finding reported in other studies (2comprising 9 FNS (fluconazole MIC, 4 to 64 μg/ml0). On the other hand, fluconazole-exposed strains did not overexpress the *CDR1* gene. This result coincides with other analyses showing that the ABC family transporters are nonspecific and are not-determinant in conferring resistance to fluconazole in *C. tropicalis* species ^24^.

Concerning the results obtained for the eight strains classified as resistant, we identified overexpression of the *CDR1* gene in one strain of the *MDR1* gene in one strain, of the *ERG11* gene in six strains, and of the *ERG3* gene in four strains. Overexpression of the *MDR1* gene in the resistant strain had the highest relative value found in this study and interestingly, in this same strain, there was no overexpression of any other genes. This finding can indicate the function of *MDR1* as an independent molecular factor that does not need the overexpression of other genes to confer resistance to fluconazole.

Although some studies report overexpression of both *MDR1* and *ERG11* in resistant strains ^25^^-^^27^, others suggested that the *MDR1* gene, coding for efflux transporters of the MFS family, is a specific fluconazole-transporter among other azole drugs and can be sufficient to independently generate resistance to this antifungal drug (20which was confirmed by determination of MICs. Considering the relationship between azole susceptibility and the respiration reported for other yeast species, the respiratory activity of this isolate was investigated. Flow cytometry using rhodamine 123 and oxygraphy demonstrated an increased respiratory activity, which was not linked to an overexpression or increased number of copies of the mitochondrial genome. Among previously described resistance mechanisms, an increased activity of efflux pumps was investigated by flow cytometry using rhodamine 6G. However, the efflux of rhodamine 6G was lower in the resistant isolate than in susceptible ones. Likewise, real-time reverse transcription-PCR quantification of the expression of *C. tropicalis MDR1* (CtMDR1). Previous studies reported this particular finding for *C. albicans*^24^, and considering the virulence similarities between *C. albicans* and *C. tropicalis*, we hypothesize about the possible similitude between the azole-resistance mechanisms of these two species ^18^.

In this study, the most frequently overexpressed genes in resistant strains were *ERG11* and *ERG3*, as other researchers also indicated ^20^. This molecular phenomenon of azole resistance is likely promoted by the long and repeated exposure to this type of antifungal or by the static mechanism of action on the ergosterol biosynthesis pathway. The study by Jiang *et al*. reported that efflux transporters are not a vital mechanism to confers azolesresistance to *C. tropicalis* and, in this sense, others would be more closely related ^18^.

Half of the fluconazole-exposed and resistant strains overexpressed both the *ERG11* and *ERG3* genes. Some studies described it as a mechanism contributing to azole resistance in *Candida* spp. However, the involvement of *ERG3* overexpression as a unique mechanism in the development of resistance has been poorly described for *C. tropicalis*^26^^,^^27^. A study reported that *C. tropicalis* strains with a loss-of-function mutation in *ERG11* can only survive if a mutation in ERG3 is also present ^28^. Therefore, it is plausible that syncronic overexpression of both genes be a complementary molecular mechanism involved in the fluconazole-resistance phenomenon.

As a noteworthy finding, the resistant strain and fluconazole-exposed 23-1 had a higher overexpression of *ERG11* than the other strains. As a differentiating element, it did not have overexpression of *ERG3*. Some studies reported that *ERG11* overexpression may be the most frequent mechanism of azole resistance, but this does not necessarily imply overexpression of *ERG3*^22^^,^^29^^,^^30^. On the other hand, the lack of *ERG3* overexpression could somehow favor the overexpression of *ERG11* to survive against fluconazole. The scientific community could investigate this hypothesis to clarify and deepen the understanding of these specific resistance mechanisms associated with the *C. tropicalis* species.

The biological and molecular mechanisms associated with *Candida* spp. resistance to the pharmacological agent fluconazole are multiple and variable ^31^^-^^33^. In our region, this antifungal and others of the pharmacological group of azoles are regularly used. Therefore, it is essential to promote research that expands and elucidates the mechanisms associated with resistance. Several researchers have documented mutations in the *ERG11* and *ERG3* genes. Currently, this manuscript’s authors are developing a project in which *ERG11* will be sequenced to evaluate its mutations, and *ERG3* sequencing is intended in the future. We expect this type of research provides the regional scientific and clinical community with considerable elements for early screening of resistant strains to make effective intervention decisions.
